# Effects of Pulsed Radiofrequency Current and Thermal Condition on the Expression of β-Endorphin in Human Monocytic Cells

**DOI:** 10.3390/neurosci6030067

**Published:** 2025-07-21

**Authors:** Akira Nishioka, Toshiharu Azma, Tsutomu Mieda, Yasushi Mio

**Affiliations:** 1 Department of Anesthesiology and Pain Medicine, Kohnodai Hospital, National Center for Global Health and Medicine, Ichikawa 272-8516, Japan; 2 Anesthesiology and Perioperative Medicine, The Jikei University Graduate School of Medicine, Tokyo 105-8461, Japan; 3 Department of Anesthesiology, Saitama Medical University Hospital, Iruma 350-0495, Japan; tmieda@saitama-med.ac.jp; 4 Department of Anesthesiology, The Jikei University School of Medicine, Tokyo 105-8461, Japan; mio@jikei.ac.jp

**Keywords:** proopiomelanocortin (POMC), beta-endorphin, monocytes, THP-1 cells, pulsed radiofrequency current, thermal effect, apoptosis, neuropathic pain

## Abstract

Pulsed radiofrequency (PRF) current applied to peripheral nerves is a modality used in interventional pain medicine, but its underlying mechanisms remain unclear. This study aimed to investigate whether ex vivo exposure of human monocytic THP-1 cells to PRF current or to heat induces β-endorphin production. Methods: THP-1 cells were exposed to PRF current for 15 min or incubated at elevated temperatures (42 °C to 50 °C) for 3 or 15 min. Flow cytometry was used to assess cell viability, and β-endorphin concentrations in culture supernatants were quantified by ELISA. In a separate experiment, cells were stimulated with lipopolysaccharide (LPS) to compare its effects on β-endorphin release. Results: A 3 min exposure to temperatures ≥ 46 °C reduced THP-1 cell viability, whereas a 15 min exposure to PRF current or to heat at 42 °C did not impair viability. Both PRF current and mild heat significantly enhanced β-endorphin release. β-Endorphin levels in the supernatant of LPS-stimulated cells were comparable to those of cells exposed to PRF current. Conclusions: Ex vivo application of PRF current or mild heat enhanced β-endorphin production from THP-1 cells without significant cytotoxicity. These preliminary findings warrant further investigation using primary human monocytes and in vivo models to assess therapeutic potential.

## 1. Introduction

Recruitment of circulating immune cells, including monocytes, to inflamed tissues has been demonstrated in experimental models of nociceptive pain, such as those induced by inoculation with Freund’s complete adjuvant (FCA), which causes chronic inflammation [1]. Several lines of evidence indicate that these recruited immune cells contribute to antinociception through enhanced production of endogenous opioids within inflamed tissues. Endogenous opioids derived from immune cells—primarily monocytes and macrophages—are also suggested to contribute to antinociception in experimental models of neuropathic pain, such as chronic constriction injury (CCI) of the sciatic nerve [2]. Peripheral transfusion of monocytes/macrophages pretreated ex vivo to produce endogenous opioids has been investigated as a potential therapeutic strategy for nerve injury [3]. However, the effects of pulsed radiofrequency (PRF) current on such ex vivo treatment in the context of neuropathic pain have not yet been evaluated.

PRF and continuous radiofrequency (CRF) are two clinical modalities widely used for the management of chronic pain [4,5,6]. CRF was originally developed to thermally ablate nerve tissue through the continuous application of a high-frequency current, typically around 500 kHz, resulting in sustained tissue heating around the electrode tip. To prevent excessive thermal damage, CRF devices are equipped with temperature control systems that regulate the tip temperature, often maintaining it at 70–90 °C during treatment. In contrast, PRF delivers short bursts of RF current (typically 20 ms at 2 Hz), which markedly limits tissue heating while still applying high peak voltages. This intermittent stimulation leads to a reduced average power delivery—approximately 4% of that in CRF at the same voltage—thus minimizing thermal injury [4,5]. Importantly, PRF has been shown to modulate neural and immune function without causing neurodestructive effects, and clinical evidence supports its efficacy in treating various neuropathic pain conditions, such as postherpetic neuralgia [7,8], cervical radicular pain [9], or chronic knee osteoarthritis [10]. Despite these findings, the underlying cellular mechanisms influenced by PRF, particularly those involving immune cells like monocytes and macrophages, remain incompletely understood.

We previously demonstrated that exposure of human monocytic THP-1 cells to a PRF electric field increased mRNA expression of proopiomelanocortin (POMC), the precursor of β-endorphin, and found that thermal conditions alone, even in the absence of PRF current, were responsible for this upregulation [5]. Interestingly, we also observed a significant increase in POMC mRNA expression under hypothermic conditions, in which THP-1 cells sedimented in test tubes were exposed to PRF current while being incubated at 20 °C [5]. These findings suggest that PRF may enhance POMC gene expression through mechanisms independent of thermal effects.

β-Endorphin is produced as part of the amino acid sequence of the precursor protein POMC, which also gives rise to several other bioactive peptides in the pituitary gland, melanocytes, and immune cells, including monocytes [11,12]. Post-translational processing of POMC by tissue-specific proteases leads to the production of various hormones and bioactive compounds, such as adrenocorticotropic hormone (ACTH), lipotropins, melanocyte-stimulating hormones (MSHs), corticotropin-like intermediate peptide (CLIP), and β-endorphin. Among these, ACTH and β-lipotropin undergo further cleavage to generate smaller peptides, including α-MSH and CLIP from ACTH, and β-MSH and β-endorphin from β-lipotropin [11]. Because β-endorphin is one of the key molecules responsible for immune cell-derived antinociception and is known to be produced by monocytes, we hypothesized that exposure of human monocytic cells to PRF current and/or heat would increase β-endorphin expression at the protein level, as we previously observed at the mRNA level.

There is mounting experimental evidence that PRF induces neuromodulation via electric field-dependent increases in intracellular Ca^2+^ levels. For instance, Mercadal et al. demonstrated that exposure of HEK-293 cells to electric fields generated by PRF current-elevated intracellular Ca^2+^ concentrations in a field strength-dependent manner [13]. Meanwhile, Sauer et al. reported that transient stimulation of human monocytes with lipopolysaccharide (LPS) promotes β-endorphin release via Toll-like receptor 4 and that this release requires elevated intracellular Ca^2+^ levels [14]. These findings may suggest a convergence in mechanism, whereby PRF may similarly enhance endogenous opioid production in monocytes by modulating intracellular Ca^2+^ dynamics. However, the transcriptional upregulation and time course observed in our study [5] imply that PRF-induced β-endorphin production may also reflect a sustained cellular response rather than merely transient peptide release.

Given the capacity of monocytes to produce β-endorphin and their established role in peripheral antinociception, there is growing interest in harnessing these cells as therapeutic vehicles. A previous study by Pannell et al. has explored the transfusion of monocytes/macrophages pretreated ex vivo to enhance opioid peptide production as a potential approach for pain management [3]. In this context, our study serves as a foundational investigation into the ability of PRF and thermal modulation to upregulate β-endorphin production in monocytic cells under controlled conditions. The findings may provide a basis for future development of ex vivo monocyte-based therapies for neuropathic pain.

## 2. Methods

### 2.1. Materials

Tetramethylrhodamine methyl ester (TMRM) was obtained from AAT Bioquest (Pleasanton, CA, USA). Fetal bovine serum (FBS) was purchased from Biowest (Nuaillé, France). UltraGlutamine (alanyl-L-glutamine) was from Lonza (Basel, Switzerland). CountBright absolute counting beads (approx. 7 µm in diameter) were from Molecular Probes (Eugene, OR, USA). Annexin V-633 was obtained from Nacalai Tesque (Kyoto, Japan). The Human Beta-Endorphin ELISA Kit (Colorimetric) was from Novus Biologicals (Minneapolis, MN, USA). RPMI-1640 HEPES Modification medium (R5886, with 25 mM HEPES, without L-glutamine) was purchased from Sigma-Aldrich (St. Louis, MO, USA).

The culture medium used in this study was RPMI-1640 containing phenol red and 25 mM HEPES. It was supplemented with 2 mM UltraGlutamine, 1% (*v*/*v*) Pen Strep, and 10% (*v*/*v*) FBS to prepare the complete culture medium. All chemicals used were certified for molecular biology or cell culture. All solutions were membrane-filtered before storage to minimize background noise in flow cytometry.

### 2.2. THP-1 Cells

The human monocytic cell line THP-1, originally provided by the American Type Culture Collection, was purchased from DS Pharma Biomedical (Suita, Osaka, Japan). Cells were cultured at a density of 1–9 × 10^5^ cells/mL in 25-cm^2^ plastic cell culture flasks filled with the complete culture medium, in a humidified atmosphere of 5% CO_2_ in air at 37 °C.

### 2.3. General Experimental Protocol

Experiments were performed according to previously described methods with modifications [5,6]. THP-1 cells were washed three times by centrifugation at 200× *g* for 2 min using the complete culture medium. Cell counts were performed twice using the automated cell counter TC20 (Bio-Rad, Hercules, CA, USA), and the average value was used to resuspend the cells at pre-selected densities in the complete culture medium.

THP-1 cell suspensions (1 × 10^6^ cells in 100 μL) were transferred to PCR tubes and incubated using a MiniAmp Plus Thermal Cycler (Thermo Fisher Scientific, Waltham, MA, USA). After a 60 min incubation at 37 °C, the cells were further incubated for 3 or 15 min at various temperatures (37 °C–50 °C). After this incubation, the cells were diluted with the complete culture medium to a final density of 5 × 10^5^ cells/mL in 24-well culture dishes and cultured for 24 h in a humidified atmosphere containing 5% CO_2_ in air.

In parallel, a separate cell suspension (1 × 10^6^ cells in 1000 μL) in a 15 mL polypropylene conical tube was centrifuged at 1000× *g* for 2 min. A radiofrequency (RF) probe with a 10 cm shaft and 4 mm active tip guiding needle (22-gauge; Hakko, Chikuma, Nagano, Japan) was inserted into the tube, positioning the active tip in the sedimented THP-1 cells. A counter electrode was attached to the plastic insulation of the guiding needle as previously described [6]. RF current at 480 kHz was applied to the sedimented cells for 15 min using a NeuroTherm NT500 RF generator (Abbott Laboratories, Chicago, IL, USA). During RF application, the tube was placed in a dry thermal bath at 37 °C in room air. As a control, another tube was prepared with the RF probe inserted for 15 min without current application.

The PRF mode of the NT500 system (repeated 480 kHz RF bursts for 20 ms at 2 Hz) is programmed to maintain a constant voltage at a pre-selected level while keeping the probe temperature below 43 °C [5,6]. When the probe temperature approached 43 °C, the system automatically prolonged the interval between RF bursts. We selected the maximum voltage (70 V) for the PRF mode and confirmed that the probe temperature remained below 43 °C throughout the procedure. After RF treatment, the cells were resuspended by gentle pipetting. Finally, all treated cell suspensions were adjusted to 5 × 10^5^ cells/mL with the complete culture medium and cultured in a humidified atmosphere of 5% CO_2_ in air for 24–48 h.

### 2.4. Flow Cytometry

At 24 h after THP-1 cells were exposed to PRF electric fields or to heat, the cells were incubated for 15 min with 10% (*vol*/*vol*) culture medium containing tetramethylrhodamine methyl ester (TMRM) and Annexin V-633 at final concentrations of 100 nM and 1% (*vol*/*vol*), respectively. A 40 µL aliquot of the cell suspension and a 4 µL aliquot of CountBright absolute counting beads (1 × 10^6^ beads/mL) were transferred to a polystyrene tube, and the volume was adjusted to 400 µL with culture medium supplemented with 2 mM CaCl_2_. Particles in the suspension were analyzed using a FACS Canto II flow cytometer (Becton Dickinson [BD], Franklin Lakes, NJ, USA), according to previously described methods [5,6].

### 2.5. Stimulation of THP-1 Cells with LPS

THP-1 cells were washed by centrifugation at 200× *g* for 2 min, following the General Experimental Protocol (Section 2.3). The cells were then resuspended in complete culture medium at a density of 3 × 10^5^ cells/mL and seeded at 1 mL per well in 24-well multiplates. These cells were incubated at 37 °C in a humidified atmosphere of 5% CO_2_ in air, either in the presence or absence of 100 ng/mL LPS. For the control group (without LPS), culture supernatants were collected at 1, 2, 4, and 6 days after incubation. These supernatants were centrifuged at 1000× *g* for 3 min and transferred to cryogenic tubes, then stored in the vapor phase of liquid nitrogen until further analysis. At 4 days of incubation, LPS-treated THP-1 cells exhibited activation and adherence to the bottom of the wells. To remove non-adherent and non-activated cells, the wells were gently rinsed with complete medium. The adherent cells were further cultured in fresh medium containing 100 ng/mL LPS. Supernatants were collected 48 h after rinsing and processed in the same manner as described above.

### 2.6. Measurement of β-Endorphin in the Supernatant of THP-1 Cells

An RF probe with a guiding needle was inserted into a 15 mL polypropylene conical tube containing sedimented THP-1 cells (1 × 10^6^ cells in 1000 μL), as described in Section 2.3. The active tip of the RF probe was positioned within the cell pellet. A counter electrode was attached to the plastic insulation of the guiding needle, and PRF was applied for 15 min. During PRF application, the tube was placed in a dry thermal bath at 37 °C under ambient air conditions. For control groups, tubes were prepared in the same manner, with RF probe insertion, and incubated at either 37 °C or 42 °C without RF current application.

After the 15 min exposure, the cells were gently resuspended by pipetting and transferred to culture plates. They were incubated at 37 °C for 48 h in a humidified atmosphere containing 5% CO_2_ in air. After incubation, the culture supernatants were collected by centrifugation at 1000× *g* for 3 min and transferred into cryogenic tubes. These samples were stored in the vapor phase above liquid nitrogen until analysis. The concentration of β-endorphin in the supernatant was measured by enzyme-linked immunosorbent assay (ELISA) using a human β-endorphin ELISA kit (Novus Biologicals), following the manufacturer’s instructions. This assay detects β-endorphin based on a colorimetric reaction in which horseradish peroxidase catalyzes the oxidation of tetramethylbenzidine (TMB), generating a color change. Optical density (OD) at 650 nm was continuously measured every 10 s for 15 min using a microplate reader Multiskan Sky (Thermo Fisher Scientific, Waltham, MA, USA). Since the β-endorphin concentrations in our samples were close to the assay’s detection limit, we verified that the OD value of the lowest standard (3.91 pg/mL) was significantly lower than that of the zero standard (Figure S1a). Sample concentrations were calculated based on a standard curve optimized for the detection range of 3.91–31.25 pg/mL (Figure S1b).

### 2.7. Statistical Analysis

Cell density was calculated based on the ratio of the number of cells to that of CountBright absolute counting beads (1 × 10^6^ beads/mL) and was expressed with a 95% confidence interval (CI). The percentage of apoptotic cells (%Apoptosis) was defined as the number of apoptotic cells divided by the total number of THP-1 cells. Comparisons of ratios or proportions between groups were performed using the chi-square test. Data obtained from ELISA or temperature measuring were expressed as mean ± standard deviation (SD). Comparisons of these data among groups were conducted using analysis of variance (ANOVA) followed by post hoc Tukey’s HSD test. A statistical difference was considered significant at a *p*-value of less than 0.05.

## 3. Results

### 3.1. Characteristics of THP-1 Cell Suspensions in Flow Cytometry

**Figure 1:**
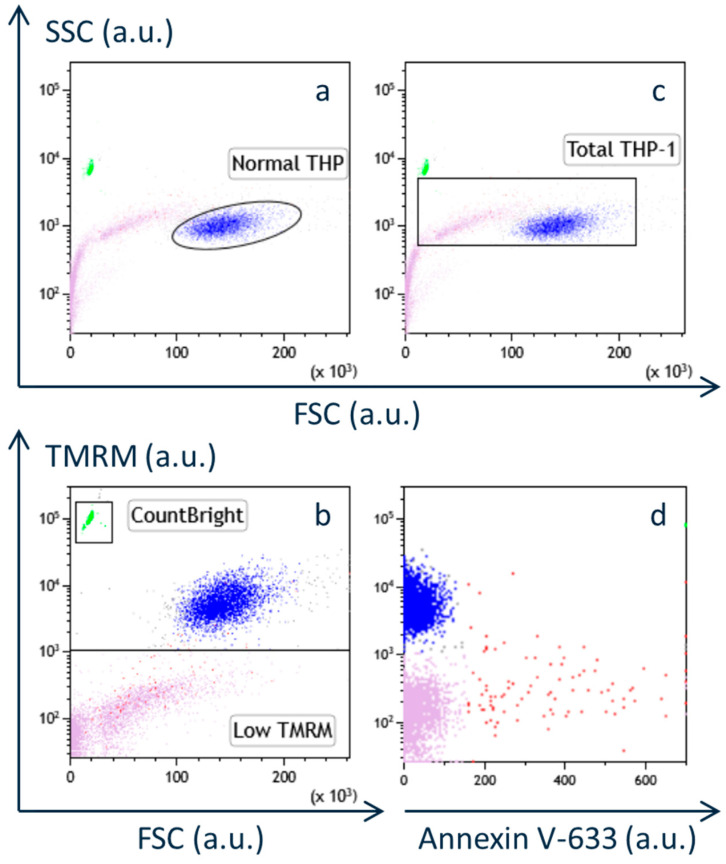
Flow cytometry analysis of suspended THP-1 cells during subculture without centrifugation at 200× *g*. (**a**) Side scatter (SSC) plotted against forward light scatter (FSC) of the cell suspension. Normal THP-1 cells are gated within the circle and colored blue. (**b**) Fluorescence intensity of tetramethylrhodamine methyl ester (TMRM) plotted against FSC of the same cell suspension as shown in (**a**). CountBright counting beads are gated in a square and colored green. Particles with low TMRM fluorescence intensity are gated in a square and shown in purple. (**c**) FSC/SSC plot of the THP-1 cell suspension with a square gate for “total THP-1”. (**d**) Fluorescence intensity of TMRM plotted against fluorescence intensity of Annexin V-633 from the same cell suspension as shown in (**c**), where only particles gated in the “total THP-1” was indicated. Particles positively stained with Annexin V-633 are colored red. (a.u., arbitrary unit).

Flow cytometric analysis was performed on THP-1 cells in suspension during subculture without centrifugation at 200× *g*. The distribution of particles was visualized in a plot of side scatter (SSC) against forward scatter (FSC) (Figure 1a). Normal THP-1 cells (gated in a circle in Figure 1a and colored blue in subsequent panels) exhibited the highest FSC values, which are generally indicative of particle size in flow cytometry [5].

The fluorescence intensity of TMRM was higher in normal THP-1 cells (blue) compared to other particles (Figure 1b). TMRM is a lipophilic, cationic fluorescent dye that accumulates in the inner mitochondrial membrane under normal membrane potential [15]. A critical loss of mitochondrial membrane potential, such as that caused by calcium overload, leads to the release of TMRM from mitochondria, thereby decreasing its fluorescence signal [15]. Thus, TMRM fluorescence reflects mitochondrial membrane potential as well as the abundance of intact mitochondria within cells or vesicles.

Particles with lower TMRM fluorescence (gated as “Low TMRM” in Figure 1b and colored purple) showed a wide range of FSC values, spanning from the detection limit of our cytometer (below 1 µm) to values approaching those of normal THP-1 cells (Figure 1a–c). Closer inspection revealed two distinct subpopulations separated by FSC values around that of CountBright absolute counting beads (7 µm; gated in a square in Figure 1b and colored green).

When the cell suspension was stained with the nuclear dye Hoechst 33342 [16], only particles with FSC values larger than the counting beads were stained, indicating that particles smaller than the beads lacked nuclei. Based on this, we defined “total THP-1 cells” as particles with FSC values between the minimum value of CountBright beads and the maximum of normal THP-1 cells, and SSC values between the minimum SSC values of normal THP-1 cells and CountBright beads (Figure 1c).

Notably, particles with low TMRM fluorescence (purple) had higher SSC values than normal THP-1 cells (blue) at comparable FSC values (Figure 1a), suggesting increased optical complexity, which may be due to surface irregularities or internal structural changes [5,16]. These features are characteristic of apoptotic cells or vesicles, which are typically smaller and more granular than healthy cells [16].

To further characterize these particles, the cell suspension was also incubated with Annexin V-633, which binds to phosphatidylserine (PS) exposed on the outer leaflet of apoptotic cells [17,18]. Annexin V-conjugated fluorophores are widely used for detecting early apoptosis [18]. In Figure 1d, the TMRM fluorescence is plotted against Annexin V-633 fluorescence for particles within the “total THP-1 cell” gate (Figure 1c). Particles stained positively with Annexin V-633 (colored red) were predominantly found within the low TMRM population. This supports the view that mitochondrial dysfunction precedes and contributes to the induction of apoptosis [18].

### 3.2. Effects of Thermal Condition and PRF Current on the Cell Viability

**Figure 2:**
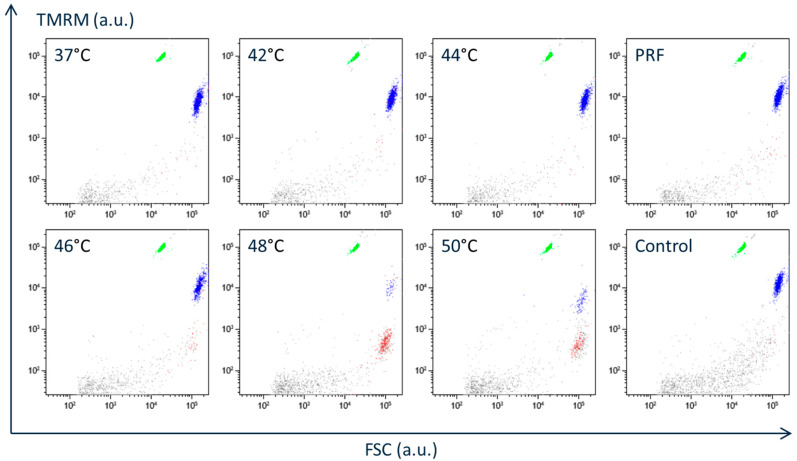
Suspension of THP-1 cells incubated at 37 °C in a humidified atmosphere of 5% CO_2_ in air for 24 h after application of 3 min heat or 15 min pulsed radiofrequency current was subjected to flow cytometry. (37 °C) Suspension of THP-1 cells was incubated at 37 °C for 3 min, and the culture continued at 37 °C for 24 h in a humidified atmosphere of 5% CO_2_ in air. (42 °C, 44 °C, 46 °C, 48 °C, and 50 °C) Suspension of THP-1 cells was incubated for 3 min at the indicated temperatures, and the culture continued under the same conditions as in (37 °C). (PRF) The pulsed radiofrequency electric current (repeated 20 ms electric current at 480 kHz at 2 Hz) was applied to the sedimented cells for 15 min using a NeuroTherm NT500 RF generator (Abbott Laboratories, Chicago, IL, USA). After this application, the culture continued under the same conditions as in (37 °C). (Control) The electric probe for the PRF application was inserted into the cell suspension for 15 min without generating current. After this application, the culture continued under the same conditions as in (PRF). (a.u., arbitrary unit).

THP-1 cells were subjected to flow cytometry after 24 h of incubation at 37 °C following a 3 min exposure to elevated temperatures (Figure 2). The density of normal THP-1 cells maintained at 37 °C throughout the experiment was 1.79 (1.62–1.97) × 10^5^ cells/mL (Table 1), where “normal THP-1 cells” were particles enclosed within a predefined gate in the FSC/SSC plot (Figure 1a). It should also be noted that the number of particles other than normal THP-1 cells was lower in Figure 2 than in Figure 1, because the THP-1 cell suspensions in Figure 2 were washed by centrifugation at 200× *g*, as described in the Section 2 under the “General Experimental Protocol”. A 3 min incubation at 42 °C or 44 °C did not significantly affect the cell density. However, exposure to 46 °C or higher temperatures resulted in a marked decrease in the density of normal THP-1 cells.

The proportion of apoptotic cells, identified by positive Annexin V-633 staining and colored red in Figure 2, remained unchanged after incubation at 42 °C or 44 °C compared to that observed at 37 °C. In contrast, exposure to 46 °C or higher significantly increased the proportion of apoptotic cells (Figure 2 and Table 1).

Application of PRF current for 15 min had no significant effect on either the density of normal THP-1 cells or the proportion of apoptotic cells (Figure 2 and Table 1). As described in the Methods, the present approach to applying PRF current increases the temperature around the active tip to just below 43 °C. These findings therefore indicate that incubation of THP-1 cells at temperatures below 43 °C for up to 15 min does not affect either cell number or the rate of apoptosis.

### 3.3. Production of β-Endorphin in the Supernatant of THP-1 Cells

**Figure 3:**
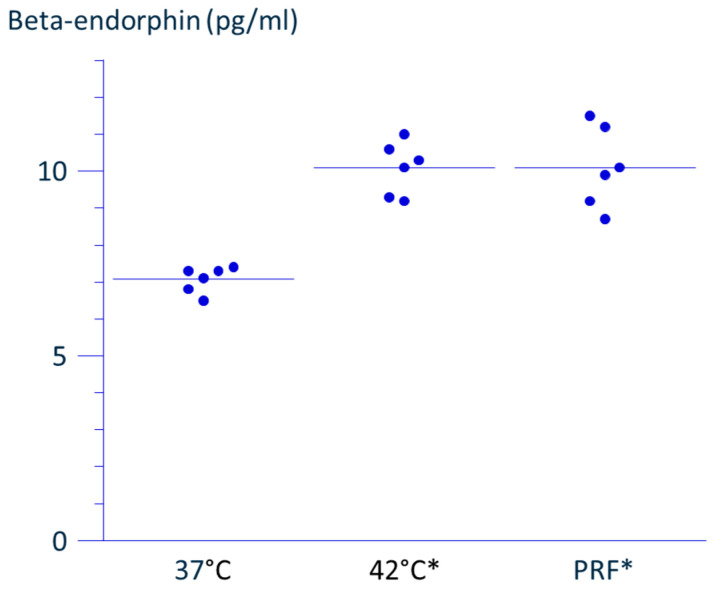
(PRF) Pulsed radiofrequency (PRF) current was applied for 15 min with a radiofrequency probe with a guiding needle, inserted into a 15 mL polypropylene conical tube containing sedimented THP-1 cells. During PRF application, the tube was placed in a dry thermal bath at 37 °C under ambient air conditions. (37 °C or 42 °C) Tubes were prepared in the same manner, with RF probe insertion, and incubated at either 37 °C or 42 °C without RF current application. Following treatment, cells were resuspended and incubated for 48 h at 37 °C. The concentration of β-endorphin in the supernatant of THP-1 cells was 7.1 ± 0.4 pg/mL in the 37 °C group, 10.1 ± 0.7 pg/mL in the 42 °C group, and 10.1 ± 1.1 pg/mL in the PRF group (mean ± SD, *n* = 6). Six individual data distribution and the mean of each group are presented in the graph. * Significantly different from the 37 °C group (*p* < 0.0001).

The concentration of β-endorphin in the supernatant collected 48 h after a 15 min incubation at 42 °C was significantly higher than that of the control group maintained at 37 °C throughout. Similarly, application of PRF current for 15 min also resulted in an increased concentration of β-endorphin in the supernatant compared to the control maintained at 37 °C throughout (Figure 3).

### 3.4. Changes in Temperature During PRF Application

The RF generator used in this study (NT500) monitors the temperature at the electrode tip of the RF probe in real time. However, because the electric field generated at the tip of the 480 kHz RF probe is highly localized, heat production is confined to only a few millimeters around the probe tip. Consequently, the overall temperature of the cell pellet, sedimented at the bottom of a conical tube containing 1 mL of culture medium with high heat capacity, may not reach the same temperature as the probe tip.

Inserting a thermocouple probe into the conical tube to monitor the temperature often led to bacterial contamination during the subsequent incubation of cells treated with the RF and thermocouple probes, due to the complexity of the procedure. Therefore, we monitored the temperature at the probe tip using the NT500 device and confirmed in real time that the temperature remained below 43 °C throughout PRF application.

To determine the actual thermal conditions near the cell pellet, we reproduced the experimental setup used in PRF exposure: the RF probe was inserted into a conical tube containing centrifuged THP-1 cell suspension, with the active tip positioned in the sedimented cells. A thermocouple probe was placed near the cell pellet, and the temperature was recorded at 1 min intervals using a digital multimeter (PC7000; Sanwa Electric Instrument, Tokyo, Japan). This experiment was repeated six times independently, yielding reproducible temperature profiles (Figure S2).

In both the 37 °C and PRF groups, the temperature reached a plateau within 2 min, while in the 42 °C group it plateaued within 3 min. Although the temperatures in both the PRF and 42 °C groups were significantly higher than those in the 37 °C group, the increase observed in the PRF group was significantly lower than that in the 42 °C group, indicating that PRF exposure caused only modest heating under the present experimental conditions.

### 3.5. Production of β-Endorphin in the Supernatant of THP-1 Cells Stimulated with LPS

LPS is a known activator of monocytes that induces β-endorphin production [14]. To evaluate the magnitude of PRF-induced β-endorphin production, we measured the concentration of β-endorphin in the supernatant of THP-1 cells stimulated with LPS using ELISA.

**Figure 4:**
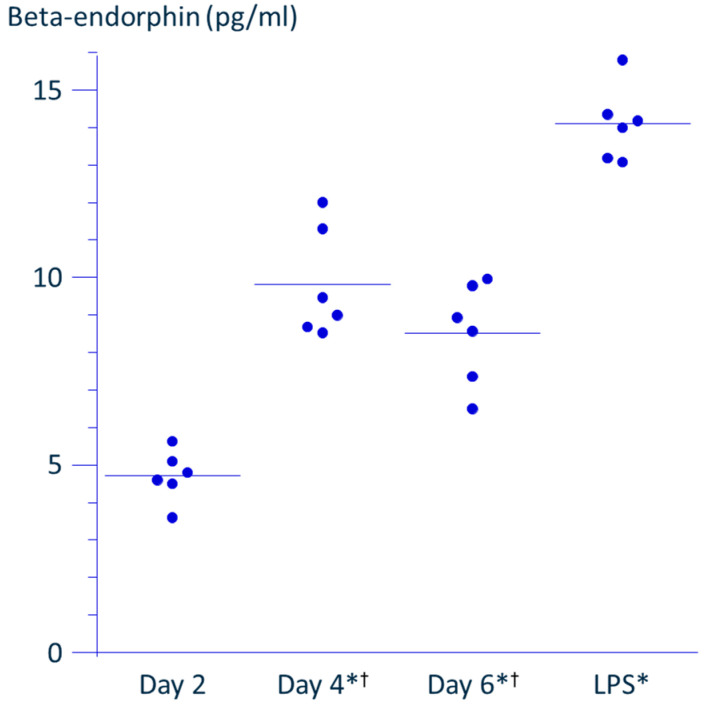
THP-1 cells were incubated in the absence or presence of 100 ng/mL lipopolysaccharide (LPS). Culture supernatants were collected on 1, 2, 4, and 6 days after incubation without LPS, and 48 h after medium replacement on day 4 for the LPS-stimulated cells. Note that the concentration of β-endorphin collected on day 1 was under the detection limit of the ELISA system. The β-endorphin concentration in the supernatant collected on day 2 was 4.7 ± 0.9 pg/mL, on day 4 was 9.1 ± 0.4 pg/mL, and on day 6 was 8.7 ± 1.1 pg/mL. The β-endorphin concentration in the supernatant collected at 48 h after the replacement of culture medium of THP-1 cells stimulated with LPS for 4 days was 14.2 ± 1.0 pg/mL (mean ± SD, *n* = 6). Six individual data distribution and the mean of each group are presented in the graph. * Significantly different from Day 2 (*p* < 0.0001). ^†^ Significantly different from LPS (*p* < 0.0001).

THP-1 cells were seeded at a density of 3 × 10^5^ cells/mL (1 mL per well) in 24-well multiplates and incubated at 37 °C in a humidified atmosphere of 5% CO_2_ in air. Supernatants were collected from separate wells at 1, 2, 4, and 6 days after incubation and compared to those from PRF-treated cells. At day 1, the β-endorphin concentration in the supernatant was below the detection limit of the ELISA system. As THP-1 is a proliferative cell line, the cell density increased over time, reaching just under 1 × 10^6^ cells/mL by day 4. Since the recommended density range for culturing THP-1 cells is 1 × 10^5^–1 × 10^6^ cells/mL, the β-endorphin concentration in the supernatant peaked at day 4 (Figure 4).

Upon stimulation with 100 ng/mL LPS, THP-1 cells adhered to the culture wells and ceased proliferation during the 4-day incubation period. To assess β-endorphin accumulation in this adherent phase, supernatants were collected 48 h after rinsing the LPS-loaded wells. The β-endorphin concentration in the supernatant of LPS-stimulated cells was 1.64-fold higher than the peak concentration observed in the unstimulated control.

## 4. Discussion

We have previously reported that exposure of human monocytic THP-1 cells to a PRF electric field for 15 min using the NT500 RF generator, which maintains the temperature around the electrode tip below 43 °C, increased mRNA expression of POMC, compared with non-exposed cells [5]. In the present study, we confirmed that PRF application also enhanced the extracellular release of β-endorphin, a final bioactive product derived from POMC, as shown by ELISA.

Several earlier studies have demonstrated that leukocytes, including monocytes, can produce endogenous opioids in inflamed tissues or around injured peripheral nerves. Rittner et al. showed in a rat model of chronic inflammatory pain induced by Freund’s complete adjuvant (FCA) inoculation that granulocytes expressing opioids were recruited to inflamed tissue and regional lymph nodes in the early phase of inflammation [1]. These granulocytes were later replaced by monocytes and macrophages as the dominant opioid-producing leukocytes. Analgesia, evaluated by pressure threshold after cold water swim stress, a surrogate of opioid-mediated pain relief, was enhanced in the affected limb. Similarly, Labuz et al. demonstrated that opioid-containing immune cells, including monocytes, accumulated at sites of nerve injury in a chronic constriction injury (CCI) model [2]. They further showed that these cells expressed corticotropin-releasing factor (CRF) receptors, and that local CRF injection suppressed mechanical hyperalgesia in a naloxone-reversible manner, indicating a role for leukocyte-derived opioids in CRF-induced analgesia [2]. Machelska et al. extended this concept by delivering vectors carrying the POMC gene into the inflamed paw in the FCA model, thereby promoting local production of endogenous opioids [19]. Although promising, this gene therapy approach has not been widely adopted due to limitations in reliability and feasibility. In a follow-up study, the same group stimulated murine bone marrow-derived monocytes to differentiate into macrophages ex vivo, and polarized them into M1 or M2 phenotypes. They found that M2 macrophages, but not M0 or M1, contained higher levels of intracellular opioid peptides and attenuated tactile allodynia in the CCI model [3].

Our study sought to investigate whether stimulation of human monocytic cells with PRF current, under thermally controlled conditions, could enhance β-endorphin production without inducing cell injury. In the field of interventional pain medicine, conventional RF (i.e., continuous RF current) is used to ablate peripheral nerves via thermal coagulation [20]. In the mid-1990s, PRF was introduced as a less-destructive alternative, delivering 20 ms bursts of RF current at 2 Hz [4,20,21]. Because RF-induced heat is proportional to the square of the voltage and the duration of exposure, PRF greatly reduces heat accumulation—down to 4% compared to continuous RF—allowing higher voltage use without causing thermal injury [4].

The clinical efficacy of PRF has been supported by meta-analyses, particularly in the treatment of postherpetic neuralgia (PHN) involving the trigeminal nerve [7], as well as in the cervical, thoracic, and lumbosacral regions [8], and in cases of cervical radiculopathy [9]. A randomized controlled trial also demonstrated that PRF combined with exercise provided superior pain relief for patients with chronic knee osteoarthritis compared to exercise alone [10].

In contrast to the growing body of clinical evidence supporting the use of PRF to alleviate pain in various conditions, its exact mechanism of action remains unclear. Our initial goal was to clarify whether β-endorphin production in monocytes contributes to the clinical efficacy of PRF, which is typically delivered via an electrode placed near injured peripheral nerves in patients with neuropathic pain or around regional nerves in those with osteoarthritis. However, our interest has gradually shifted toward exploring whether monocytes stimulated ex vivo with PRF might exert antinociceptive effects in vivo through β-endorphin release.

Other strategies to induce opioid production in monocytes, such as POMC gene transduction [19] or cytokine stimulation [3], pose ethical or technical challenges for clinical application. PRF, on the other hand, has been used safely for more than 30 years in clinical settings [4,20,21]. Therefore, our model—applying PRF current to suspended human monocytes ex vivo—may represent a more practical and ethically acceptable approach to promoting endogenous opioid release for pain management.

We used the Abbott NT500 RF generator, which limits the electrode tip temperature to below 43 °C. A newer Abbott device, the Ionic RF generator, allows this temperature to rise up to 49 °C. However, the classical PRF protocol (e.g., 45 V for 2 min at 42 °C [22]) lacks safety data at higher temperatures. Therefore, in this study, we first confirmed the safety of heat-only conditions (i.e., without PRF current) for up to 3 min at temperatures below 46 °C, in alignment with conventional PRF parameters. Under these conditions, no decrease in viable cell number or apoptosis was observed, supporting our earlier finding that classical PRF does not cause cytotoxicity [5,6].

In contrast, the duration of PRF exposure in our current study was extended to 15 min, consistent with our previous work [5]. This protocol was based on expert opinion from pain physicians, suggesting enhanced analgesia with longer PRF application. The NT500 generator suspends PRF delivery when the tissue temperature exceeds 42 °C, resuming only when it drops below this threshold. As a result, when temperatures rise above 42 °C, it takes more time to deliver the same cumulative energy. To address this limitation, the newer Ionic RF generator offers a protocol in which the total number of 20 ms RF bursts can be selected, rather than relying solely on a fixed duration of application.

Taken together with our previous findings, the current results indicate that applying 70 V of PRF current for 15 min at just under 43 °C enhances POMC mRNA [5], and increases β-endorphin release in THP-1 cells, without inducing cytotoxic effects [5,6].

Limitation of this study:

- Is PRF-Induced β-Endorphin Production Sufficient for Analgesia? A Comparison with LPS Stimulation

We confirmed that PRF current and heat stress enhanced the expression of β-endorphin from monocytic cells in vitro. Although previous studies have suggested that monocyte-derived β-endorphin contributes to the alleviation of neuropathic pain [1,2,3], it remains unclear whether β-endorphin production induced by ex vivo PRF stimulation of monocytic cells is sufficient to alleviate pain in patients with neuropathic or nociceptive pain.

To address this issue, we compared the effects of PRF application with those of LPS, a known inducer of β-endorphin production from monocytes. Since THP-1 cells are proliferative and undergo macrophage-like differentiation upon LPS activation, incubation of cells was adjusted so that the cell density reached the permissible maximum by day 4, corresponding to the time when LPS-induced activation is completed. This approach allowed us to match the cell density as closely as possible to that in the PRF application experiments. As shown in Figure 4, the concentration of β-endorphin in the culture supernatant peaked on day 4, suggesting that the cells had reached confluence. On day 4, the LPS-stimulated wells were rinsed and fresh medium was added; the accumulation of β-endorphin over the subsequent 48 h was then measured to match the PRF experiment protocol. The concentration of β-endorphin induced by LPS stimulation was 1.6-fold higher than the maximal level observed in the absence of stimulation, while PRF application induced a 1.4-fold increase over control. These findings suggest that PRF stimulation leads to a comparable enhancement of β-endorphin production to that induced by LPS.

Exploring β-endorphin production across different cell densities might have strengthened the consistency of the findings. However, the cell density used was already near the upper limit for THP-1 culture, while the amount of β-endorphin in the supernatant was close to the detection limit of the ELISA. This technical limitation made such evaluation difficult. Similarly, investigating the time course of β-endorphin production after PRF exposure could provide insights into the duration of PRF’s effects. However, due to the proliferative nature of THP-1 cells, prolonged observation would likely exceed optimal culture conditions.

Sauer et al. demonstrated that intraplantar injection of LPS in a CFA-induced pain model in rats produced antinociception via M2 macrophages, and this effect was antagonized by naloxone [14]. In the same study, LPS stimulation also increased β-endorphin production from monocytes in vitro. Taken together, these findings suggest that the β-endorphin produced by monocytes in response to LPS contributes to the analgesic effects observed in the CFA model, and that the levels produced are sufficient for meaningful pain relief. Based on this analogy, the PRF-induced enhancement of β-endorphin production from monocytic cells ex vivo may also represent a promising mechanism of analgesia.

- Unconfirmed Thermal Profile During the Exposure of THP-1 Cells to PRF Current

As suggested by theoretical estimations, including those by Cosman et al., high-intensity electric fields are generated in the vicinity of the RF electrode [23]. Although the electric field itself does not directly generate heat, higher field strength increases current density within the surrounding tissues, leading to localized, time-dependent temperature elevation via Joule heating (I^2^R or V^2^/R) [4,23]. Since the electric field strength decays inversely proportional to the square of the distance, thermal effects at regions farther from the electrode tip are expected to decrease accordingly. Both theoretical modeling [23] and experimental data using egg white (albumen) [24] have demonstrated that, when RF currents near 500 kHz are applied, the critical region of temperature elevation does not exceed 2–3 mm from the shaft of the electrode. In albumen-based models, when the PRF temperature setting was raised above 60 °C, visible thermal coagulation occurred; even at a maximum setting of 70 °C, the extent of coagulation remained within 3 mm of the shaft [24].

In our own experiments, the maximum temperature of the medium surrounding the cell pellet in conical tubes during PRF application under identical conditions was 40.4 ± 0.7 °C (Figure S2). Based on these observations, it is presumed that the individual THP-1 cells sedimented in the tube were exposed to a thermal gradient ranging from 40 °C to 43 °C.

In a previous report, we demonstrated that PRF current applied to THP-1 cells in tubes pre-cooled to 20 °C led to an upregulation of POMC mRNA expression comparable to that observed during incubation at 37 °C [5]. These findings suggest that PRF stimulation may enhance β-endorphin production through mechanisms independent of thermal effects.

Mercadal et al. cultured HEK-293 cells on coverslips and loaded them with the calcium indicator dye Calcium Green-1 to monitor intracellular Ca^2+^ levels at the single-cell level [13]. When exposed to a 500 kHz PRF electric field, they observed transient increases in intracellular calcium concentrations, which were shown to correlate with the electric field strength calculated based on the distance from the electrode. These findings suggest that the cellular response to PRF varies depending on the distance from the electrode, and thus the field strength experienced by each cell. Theoretically, it is also expected that the temperature distribution within the solution is not uniform.

Based on this context, it remains uncertain whether the observed upregulation of β-endorphin production in our study was confined to a subpopulation of THP-1 cells that was exposed to temperatures approaching 42 °C, or whether PRF-induced effects also occurred in cells experiencing minimal thermal input but significant electric field exposure.

- Possible Mechanisms of Action of PRF Current in the Modulation of Cellular Function

To date, no experimental study has definitively established a mechanism by which PRF current modulates cellular function independently of thermal effects. Cosman et al. proposed that the high electric field strengths generated by clinically available RF generators may lead to membrane depolarization or even electroporation, potentially altering ion channel activity [23]. Although Mercadal et al. provided experimental support for these hypotheses [13], it should be noted—as discussed in the previous section—that local temperature elevations are inherently coupled with electric field strength due to Joule heating, and thus difficult to isolate experimentally.

Our previous literature review, along with earlier reports, comprehensively examined the neuromodulatory effects of PRF [5]. As of the present manuscript, no substantial new findings have emerged that would significantly alter the current understanding of PRF-induced cellular modulation.

However, a pathway reported in 2018 may offer a new perspective. The discovery of the itaconate-mediated intracellular protective pathway activated in monocytes and macrophages by LPS stimulation [25] may provide a potential link between PRF stimulation and cellular modulation. LPS is classically known to stimulate monocytes to produce proinflammatory cytokines such as IL-1β and IL-6 and is widely used to induce an M1 macrophage phenotype [3]. Nevertheless, LPS also upregulates the expression of immunoresponsive gene 1 (Irg1), which encodes aconitate decarboxylase 1, leading to the intracellular accumulation of itaconate [25].

Itaconate, in turn, alkylates KEAP1, resulting in the activation of the Nrf2 pathway. This activation enhances antioxidant activity in monocytes/macrophages and promotes the production of anti-inflammatory cytokines such as IL-10, thereby counteracting acute inflammatory responses [25]. Although several studies have reported that anti-inflammatory M2 macrophages produce higher levels of β-endorphin than their proinflammatory M1 counterparts, M1 macrophages are not entirely devoid of β-endorphin-producing capacity [3,14]. Moreover, spinal application of 4-octyl itaconate (4-OI), a cell-permeable derivative of itaconate, has been shown to increase endorphin activity in a chronic constriction injury (CCI) mouse model in an IL-10-dependent manner [26]. These findings suggest that itaconate pathways may serve as a regulatory axis for endogenous opioid production.

Whether PRF stimulation enhances intracellular itaconate accumulation or activates the Nrf2 pathway remains unknown. However, future investigations into the relationship between PRF exposure and itaconate metabolism may be key to elucidating the cellular mechanisms underlying PRF-induced modulation of immune cell function.

- Speculative Role of Heat Shock Proteins in PRF-Induced Cellular Effects

Mild hyperthermia generally induces the expression of heat shock proteins (HSPs), which play cytoprotective roles by refolding denatured proteins, preventing aggregation, and aiding in the degradation of irreversibly damaged proteins [27]. Given that PRF application induces mild thermal stress, it is plausible that HSP expression is also modulated during PRF exposure, potentially protecting THP-1 cells from thermal damage. Recent evidence has shown that HSPB8, upregulated in hyperthermia-treated triple-negative breast cancer (TNBC) cells, can be transferred via exosomes into THP-1 cells and modulate macrophage polarization [28]. This suggests that HSP expression induced by PRF might influence monocyte/macrophage polarization. However, while HSPs like HSPB8 may be involved in immune modulation, several studies have shown that HSP expression is predominantly upregulated in M1-polarized monocytes/macrophages, but not in M2 phenotypes [29,30]. Since M2 macrophages are considered the major producers of opioid peptides such as β-endorphin [3], the role of HSPs in PRF-induced β-endorphin production remains ambiguous. Taken together, HSPs may have multiple roles in this context, including cytoprotection, regulation of monocyte polarization, and possibly modulation of β-endorphin production. However, this study did not evaluate the expression of HSPs nor examine whether PRF alters the polarization state of monocytes. Future studies should assess cytokine profiles and HSP expression following PRF exposure to clarify these mechanisms.

- Translational Considerations and Future Directions

We used the THP-1 cell line rather than primary human monocytes to ensure a reliable supply and to avoid ethical concerns. While THP-1 cells are widely used as a model of human monocytes, future studies should include primary cells to validate the translational relevance of the findings. Finally, clinical translation of this concept requires investigator-initiated trials to determine optimal cell doses and therapeutic efficacy in humans.

This study serves as a preliminary step toward a novel therapeutic concept in which PRF-stimulated monocytes could be reintroduced near peripheral nerves to alleviate neuropathic pain—leveraging a technique already used clinically, warranting further investigation.

## 5. Conclusions

A 15 min exposure of THP-1 cells to sub-43 °C heat, as well as to pulsed radiofrequency (PRF) current, promoted the extracellular release of β-endorphin.

## Figures and Tables

**Table 1:** Effects of heat and pulsed radiofrequency current on the cytotoxicyty of THP-1 cells.

Application		Cell Count	Density of Normal THP-1 Cells			Apoptosis		
		(n)	(10^5^ cells/mL)	(95% CI)		(%)	(95% CI)	
37 °C	3 min	(1043)	1.79	(1.62–1.97)		2.49	(1.69–3.64)	
42 °C	3 min	(1029)	1.86	(1.69–2.05)	* N.S.	3.21	(2.28–4.48)	* N.S.
44 °C	3 min	(870)	1.56	(1.41–1.73)	* N.S.	2.41	(1.56–3.68)	* N.S.
46 °C	3 min	(624)	1.04	(0.94–1.16)	* *p* < 0.01	7.85	(5.98–10.25)	* *p* < 0.01
48 °C	3 min	(416)	0.69	(0.61–0.78)	* *p* < 0.01	45.9	(41.18–50.72)	* *p* < 0.01
50 °C	3 min	(395)	0.71	(0.63–0.81)	* *p* < 0.01	25.6	(21.51–30.10)	* *p* < 0.01
PRF	15 min	(1004)	1.50	(1.36–1.65)	* *p* < 0.05, ^†^ NS	3.69	(2.67–5.05)	*^†^ NS
control	15 min	(867)	1.37	(1.24–1.52)	* *p* < 0.05	3.69	(2.61–5.18)	* NS

N.S. not significant, * vs. 37 °C, ^†^ vs. control.

## Data Availability

No new data were created or analyzed in this study. Data sharing is not applicable to this article.

## Supplementary Materials

The following supporting information can be downloaded at: https://www.mdpi.com/article/10.3390/neurosci6030067/s1, Figure S1: Standard curve showing the optical density (OD) at 650 nm for samples containing known concentrations of β-endorphin; Figure S2: The actual thermal conditions near the cell pellet.

## References

[1] Rittner H.L., Brack A., Machelska H., Mousa S.A., Bauer M., Schäfer M., Stein C. (2001). Opioid Peptide-Expressing Leukocytes: Identification, Recruitment, and Simultaneously Increasing Inhibition of Inflammatory Pain. Anesthesiology.

[2] Labuz D., Schmidt Y., Schreiter A., Rittner H.L., Mousa S.A., Machelska H. (2009). Immune Cell-Derived Opioids Protect against Neuropathic Pain in Mice. J. Clin. Investig..

[3] Pannell M., Labuz D., Celik M.Ö., Keye J., Batra A., Siegmund B., Machelska H. (2016). Adoptive Transfer of M2 Macrophages Reduces Neuropathic Pain via Opioid Peptides. J. Neuroinflamm..

[4] Chua N.H.L., Vissers K.C., Sluijter M.E. (2011). Pulsed Radiofrequency Treatment in Interventional Pain Management: Mechanisms and Potential Indications-a Review. Acta Neurochir..

[5] Azma T., Nishioka A., Ogawa S., Nagasaka H., Matsumoto N. (2018). Enhanced Expression of Gene Coding for β-Endorphin in Human Monocytic Cells Exposed to Pulsed Radio Frequency Electric Fields through Thermal and Non-Thermal Effects. J. Pain Res..

[6] Nishioka A., Kimura M., Sakamoto E., Nagasaka H., Azma T. (2020). Continuous But Not Pulsed Radiofrequency Current Generated by NeuroTherm NT500 Impairs Mitochondrial Membrane Potential in Human Monocytic Cells THP-1. J. Pain Res..

[7] Wang C., Dou Z., Yan M., Wang B. (2023). Efficacy and Safety of Pulsed Radiofrequency in Herpes Zoster Related Trigeminal Neuralgia: A Systematic Review and Meta-Analysis. J. Pain Res..

[8] Wu C.-Y., Lin H.-C., Chen S.-F., Chang W.-P., Wang C.-H., Tsai J.-C., Lin Y.-C., Kao Y., Tam K.-W. (2020). Efficacy of Pulsed Radiofrequency in Herpetic Neuralgia: A Meta-Analysis of Randomized Controlled Trials. Clin. J. Pain.

[9] Kwak S.G., Lee D.G., Chang M.C. (2018). Effectiveness of Pulsed Radiofrequency Treatment on Cervical Radicular Pain: A Meta-Analysis. Medicine.

[10] Han Q., Ma Y., Jia P., Wang X., Wang B., Zheng Y. (2021). A Randomized Controlled Pilot Study Comparing the Efficacy of Pulsed Radiofrequency Combined With Exercise Versus Exercise Alone in Pain Relief and Functional Improvement for Chronic Knee Osteoarthritis. Pain Pract..

[11] Hadley M.E., Haskell-Luevano C. (1999). The Proopiomelanocortin System. Ann. N. Y. Acad. Sci..

[12] Mousa S.A., Shakibaei M., Sitte N., Schäfer M., Stein C. (2004). Subcellular Pathways of Beta-Endorphin Synthesis, Processing, and Release from Immunocytes in Inflammatory Pain. Endocrinology.

[13] Mercadal B., Vicente R., Ivorra A. (2020). Pulsed Radiofrequency for Chronic Pain: In Vitro Evidence of an Electroporation Mediated Calcium Uptake. Bioelectrochemistry.

[14] Sauer R.-S., Hackel D., Morschel L., Sahlbach H., Wang Y., Mousa S.A., Roewer N., Brack A., Rittner H.L. (2014). Toll like Receptor (TLR)-4 as a Regulator of Peripheral Endogenous Opioid-Mediated Analgesia in Inflammation. Mol. Pain.

[15] McKenzie M., Lim S.C., Duchen M.R. (2017). Simultaneous Measurement of Mitochondrial Calcium and Mitochondrial Membrane Potential in Live Cells by Fluorescent Microscopy. J. Vis. Exp..

[16] Darzynkiewicz Z., Bruno S., Del Bino G., Gorczyca W., Hotz M.A., Lassota P., Traganos F. (1992). Features of Apoptotic Cells Measured by Flow Cytometry. Cytometry.

[17] Azma T., Tuluc F., Ito T., Aoyama-Mani C., Kawahito S., Kinoshita H. (2014). Mechanisms of Action of Anesthetics for the Modulation of Perioperative Thrombosis: Evidence for Immune Mechanisms from Basic and Clinical Studies. Curr. Pharm. Des..

[18] van Engeland M., Nieland L.J., Ramaekers F.C., Schutte B., Reutelingsperger C.P. (1998). Annexin V-Affinity Assay: A Review on an Apoptosis Detection System Based on Phosphatidylserine Exposure. Cytometry.

[19] Machelska H., Schroff M., Oswald D., Binder W., Sitte N., Mousa S.A., Rittner H.L., Brack A., Labuz D., Busch M. (2009). Peripheral Non-Viral MIDGE Vector-Driven Delivery of Beta-Endorphin in Inflammatory Pain. Mol. Pain.

[20] Sluijter E.R., Cosman W.B., Rittman M., VanKleef M.E. (1998). The Effects of Pulsed Radiofrequency Fields Applied to the Dorsal Root Ganglion: A Preliminary Report. Pain Clin..

[21] Sluijter M.E., van Kleef M. (2007). Pulsed Radiofrequency. Pain Med..

[22] Van Zundert J., Brabant S., Van de Kelft E., Vercruyssen A., Van Buyten J.-P. (2003). Pulsed Radiofrequency Treatment of the Gasserian Ganglion in Patients with Idiopathic Trigeminal Neuralgia. Pain.

[23] Cosman E.R., Cosman E.R. (2005). Electric and Thermal Field Effects in Tissue around Radiofrequency Electrodes. Pain Med..

[24] Heavner J.E., Boswell M.V., Racz G.B. (2006). A Comparison of Pulsed Radiofrequency and Continuous Radiofrequency on Thermocoagulation of Egg White in Vitro. Pain Physician.

[25] Mills E.L., Ryan D.G., Prag H.A., Dikovskaya D., Menon D., Zaslona Z., Jedrychowski M.P., Costa A.S.H., Higgins M., Hams E. (2018). Itaconate Is an Anti-Inflammatory Metabolite That Activates Nrf2 via Alkylation of KEAP1. Nature.

[26] Sun Q., Hu T., Zhang Y., Wang X., Liu J., Chen W., Wei C., Liu D., Wu W., Lan T. (2022). IRG1/Itaconate Increases IL-10 Release to Alleviate Mechanical and Thermal Hypersensitivity in Mice after Nerve Injury. Front. Immunol..

[27] Morimoto R.I. (1998). Regulation of the Heat Shock Transcriptional Response: Cross Talk between a Family of Heat Shock Factors, Molecular Chaperones, and Negative Regulators. Genes Dev..

[28] Xu D., Liu Z., Liang M.-X., Chen W.-Q., Fei Y.-J., Yang S.-J., Wu Y., Zhang W., Tang J.-H. (2023). Hyperthermia Promotes M1 Polarization of Macrophages via Exosome-Mediated HSPB8 Transfer in Triple Negative Breast Cancer. Discov. Oncol..

[29] Fagone P., Di Rosa M., Palumbo M., De Gregorio C., Nicoletti F., Malaguarnera L. (2012). Modulation of Heat Shock Proteins during Macrophage Differentiation. Inflamm. Res..

[30] Zhang F., Liu H., Jiang G., Wang H., Wang X., Wang H., Fang R., Cai S., Du J. (2015). Changes in the Proteomic Profile during the Differential Polarization Status of the Human Monocyte-Derived Macrophage THP-1 Cell Line. Proteomics.

