# Supplementary material for: Effects of Pulsed Radiofrequency Current and Thermal Condition on the Expression of β-Endorphin in Human Monocytic Cells
Source: NeuroSci. 2025 Jul 21;6(3):67. doi: 10.3390/neurosci6030067 (PMC12285971; doi:10.3390/neurosci6030067)
Supplement: Supplementary file 1 [file neurosci-06-00067-s001.zip › Figure S1.pdf]

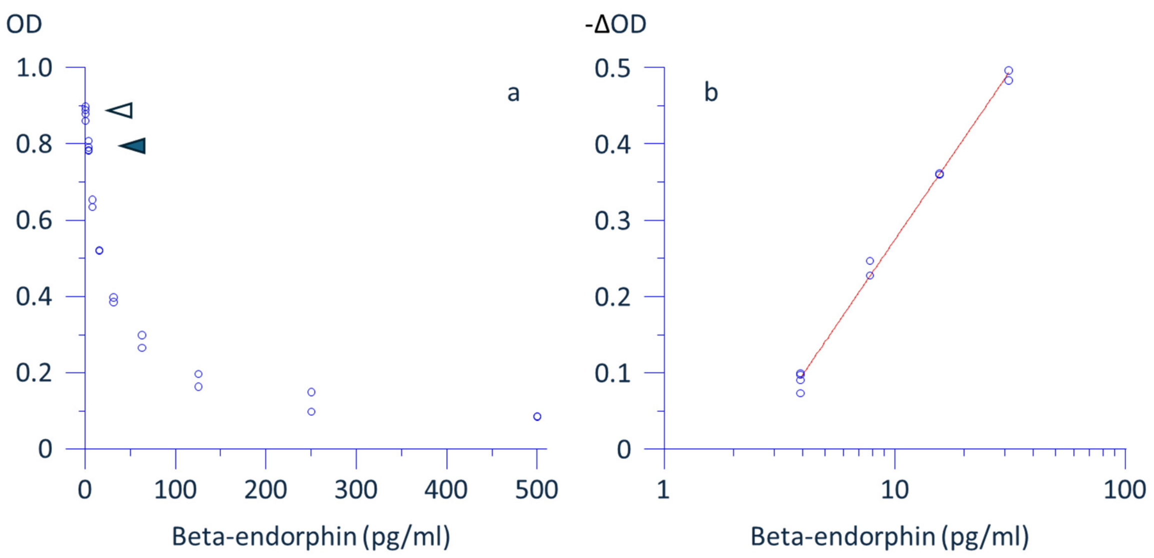

Figure S1. (a) Standard curve showing the optical density (OD) at 650 nm for samples containing known concentrations of β-endorphin, as indicated. The open arrow indicates OD values from samples without β-endorphin (n = 4), and the closed arrow indicates OD values from samples containing 3.91 pg/mL β-endorphin (n = 4). Post hoc analysis following ANOVA (Tukey's HSD test) showed a significant difference between these two groups ($P < 0.01$). (b) A refined standard curve focusing on the concentration range of 3.91 – 31.25 pg/mL. Linear regression analysis showed that ΔOD—defined as the OD of samples containing β-endorphin at 3.91, 7.81, 15.63, and 31.25 pg/mL minus the OD of β-endorphin-free samples—was significantly correlated with β-endorphin concentration ($-\Delta OD = -0.16811 + 0.4426 \times \log_{10}$[β-endorphin (pg/mL)], $R = 0.99801$).
