# Supplementary material for: Effects of Pulsed Radiofrequency Current and Thermal Condition on the Expression of β-Endorphin in Human Monocytic Cells
Source: NeuroSci. 2025 Jul 21;6(3):67. doi: 10.3390/neurosci6030067 (PMC12285971; doi:10.3390/neurosci6030067)
Supplement: Supplementary file 1 [file neurosci-06-00067-s001.zip › Figure S2.pdf]

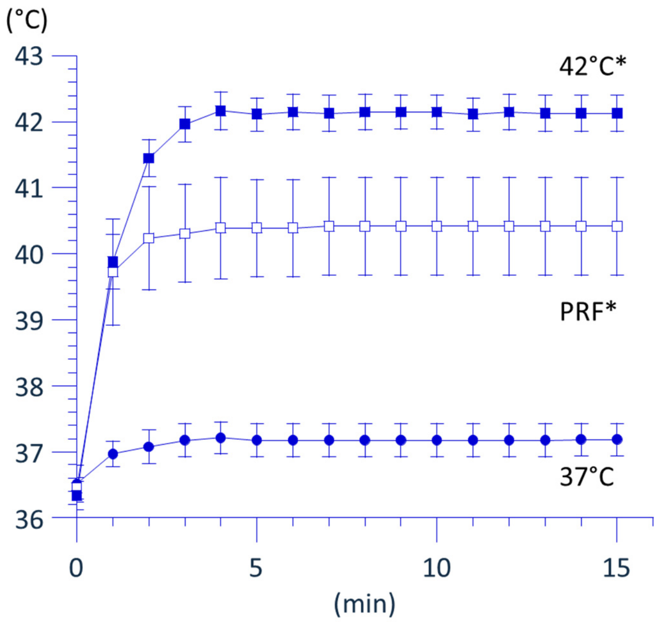

Figure S2. The actual thermal conditions near the cell pellet. The RF probe was inserted into a conical tube containing centrifuged THP-1 cell suspension, with the active tip positioned in the sedimented cells. A thermocouple probe was placed near the cell pellet, and the temperature was recorded at 1-minute intervals. Data are expressed as mean ± SD (n = 6). *Significantly different from 37°C.
